# Conservation of the *S10-spc-α* Locus within Otherwise Highly Plastic Genomes Provides Phylogenetic Insight into the Genus *Leptospira*

**DOI:** 10.1371/journal.pone.0002752

**Published:** 2008-07-16

**Authors:** Berta Victoria, Ahmed Ahmed, Richard L. Zuerner, Niyaz Ahmed, Dieter M. Bulach, Javier Quinteiro, Rudy A. Hartskeerl

**Affiliations:** 1 Department of Biochemistry and Molecular Biology, Faculty of Biology, University of Santiago de Compostela, Galicia, Spain; 2 WHO/FAO/OIE and National Collaborating Centre for Reference and Research on Leptospirosis, Department of Biomedical Research, Royal Tropical Institute (KIT), Amsterdam, The Netherlands; 3 Bacterial Diseases of Livestock Research Unit, National Animal Disease Center, Agricultural Research Service, U.S. Department of Agriculture, Ames, Iowa, United States of America; 4 Pathogen Evolution Laboratory, Centre for DNA Fingerprinting and Diagnostics, Hyderabad, India; 5 Australian Research Council Centre of Excellence in Structural and Functional Microbial Genomics, Department of Microbiology, Monash University, Clayton, Victoria, Australia; University of Liverpool, United Kingdom

## Abstract

*S10-spc-α* is a 17.5 kb cluster of 32 genes encoding ribosomal proteins. This locus has an unusual composition and organization in *Leptospira interrogans*. We demonstrate the highly conserved nature of this region among diverse *Leptospira* and show its utility as a phylogenetically informative region. Comparative analyses were performed by PCR using primer sets covering the whole locus. Correctly sized fragments were obtained by PCR from all *L. interrogans* strains tested for each primer set indicating that this locus is well conserved in this species. Few differences were detected in amplification profiles between different pathogenic species, indicating that the *S10-spc-α* locus is conserved among pathogenic *Leptospira*. In contrast, PCR analysis of this locus using DNA from saprophytic *Leptospira* species and species with an intermediate pathogenic capacity generated varied results. Sequence alignment of the *S10-spc-α* locus from two pathogenic species, *L. interrogans* and *L. borgpetersenii*, with the corresponding locus from the saprophyte *L. biflexa* serovar Patoc showed that genetic organization of this locus is well conserved within *Leptospira*. Multilocus sequence typing (MLST) of four conserved regions resulted in the construction of well-defined phylogenetic trees that help resolve questions about the interrelationships of pathogenic *Leptospira*. Based on the results of *secY* sequence analysis, we found that reliable species identification of pathogenic *Leptospira* is possible by comparative analysis of a 245 bp region commonly used as a target for diagnostic PCR for leptospirosis. Comparative analysis of *Leptospira* strains revealed that strain H6 previously classified as *L. inadai* actually belongs to the pathogenic species *L. interrogans* and that *L. meyeri* strain ICF phylogenetically co-localized with the pathogenic clusters. These findings demonstrate that the *S10-spc-α* locus is highly conserved throughout the genus and may be more useful in comparing evolution of the genus than loci studied previously.

## Introduction

Leptospirosis is one of the most widespread zoonotic diseases in the world and is caused by pathogenic spirochetes within the genus *Leptospira*. Spirochetes belong to an ancient branch of eubacteria, with *Leptospira* representing its deepest division [1]. *Leptospira* are genetically diverse bacteria. Genetic classification of this genus is based on DNA homology and divides pathogenic *Leptospira* into seven main species: *L. interrogans*, *L. borgpetersenii*, *L. weilii*, *L. noguchii*, *L. santarosai*, *L. kirschneri and L. alexanderi* [2]–[4]. In addition, there are currently eleven recognized species with a saprophytic or intermediate pathogenic status, including the saprophytic species *L. biflexa* and *L. meyeri*, and *L. fainei and L. inadai* exemplifying species with an intermediate status [5]–[9]. Whole genome sequencing of *L. interrogans* serovars Lai and Copenhageni and two strains of *L. borgpetersenii* serovar Hardjo has revealed the occurrence of frequent gene rearrangements and fragmentation, perhaps indicating a rapid adaptation to new environments by pathogenic *Leptospira* [10]–[12]. It has been proposed that genome reduction detected in *L. borgpetersenii* reflects lower environmental survivability corresponding to limited potential for indirect transmission [10], in contrast to *L. interrogans*, a species that frequently passes through surface water between mammalian hosts [13].

We previously characterized the *S10-spc-α* ribosomal protein cluster of *L. interrogans* serovar Lai [14]. The cluster consists of 17.5 kb comprising 32 genes that, with the exception of *fus*, *tuf*, *secY*, *adk* and *infA*, code for ribosomal proteins. The *secY* gene codes for preprotein translocase, a gene that has diagnostic value and potential for resolving taxonomic questions in *Leptospira* [5], [14]. Genetic organization of ribosomal proteins is highly conserved and a prototypical S10 locus may predate divergence of Archaea and Bacteria [15]. However, translocation of several genes throughout the *S10-spc-α* locus differentiates Gram-positive from Gram-negative bacteria [15]. The genetic organization of the *L. interrogans S10-spc-α* locus is unique, as it contains all genes found in the *Escherichia coli* locus, and all genes except *map* that are found in the *Bacillus subtilis* locus [14]. The *L. interrogans S10-spc-α* locus is not typical of other spirochetes; several genes found in the *S10-spc-α* locus of *L. interrogans* are translocated to different portions of the *Borrelia burgdorferi* and *Treponema pallidum* genomes [14]. Considering the high plasticity of the *Leptospira* genome [10], [11], [16], it is unclear if genetic organization of the *S10-spc-α* locus is conserved amongst *Leptospira*, or if the genetic organization shared among *Borrelia* and *Treponema* may occur among some *Leptospira* species, and predate divergence of *Leptospira* from other spirochete genera.

In this study, we examined genetic organization and content of the *S10-spc-α* locus in *Leptospira*, and report that this locus is highly conserved throughout the genus. These data suggest that maintenance of the *S10-spc-α* operon structure is essential regardless of the extent of other rearrangements that have occurred during *Leptospira* evolution. Comparative sequence analysis of four segments of the *S10-spc-α* locus provides new information on phylogenetic relationships between pathogenic *Leptospira*.

## Results

### Amplification of the S10-spc-α locus of L. interrogans

Correctly sized fragments as deduced from the positions of the primer pairs on the locus (Table 1) were obtained from all six *L. interrogans* strains (Lai, M20, RGA, Hond Utrecht IV, Pomona and Hardjoprajitno) for each of the 40 primer pairs tested. These data indicate that the *S10-spc-α* locus is well conserved in *L. interrogans* (Table S3). Remarkably, the amplification pattern of *L. inadai* serovar Malaya strain H6 was identical to that of *L. interrogans*, a finding that we note below indicates this strain was incorrectly classified previously as *L. inadai*.

**Table 1:** Primer pairs and positions in the *S10-spc-α* locus of *L. interrogans* serovar Lai.

Fragment	Primer pair	Position	Genes	Fragment	Primer pair	Position	Genes
1.	737-745	843-1435	urp	35.*	301-258	11601-12581	rplE-rplF
2.	740-744	1305-1873	urp	36.*	301-191	11601-12948	rplE-rplF
3.	748-751	1759-2493	fus	37.	314-191	12348-12948	rpsH, rplF
4. *	752-751	2269-2493	fus	38.	314-428c	12348-14047	rpsH-rpmD
5. *	752-729	2269-2832	fus	39.	314-430c	12348-14372	rpsH-rplO
6. *	735-729	2406-2832	fus	40.	802-R1c	12735-13445	rplF, rplR
7. *	735-743	2406-3304	fus	41.*	802-428c	12735-14047	rplF-rpmD
8.	735-667	2406-4394	fus, tuf	42.*	802-430c	12735-14372	rplF-rplO
9. *	743c-706	3304-3814	fus	43.*	191c-428c	12948-14047	rplF-rpmD
10.*	743c-667	3304-4394	fus, tuf	44.	191c-430c	12948-14372	rplF-rplO
11.*	800-660	3683-4327	fus, tuf	45.*	R1-428c	13427-14047	rplR-rpmD
12.	800-667	3683-4394	fus, tuf	46.	R1-430c	13427-14372	rplR-rplO
13.*	657-654	4350-5255	tuf	47.*	428-430c	14047-14372	rpmD, rplO
14.	657-624c	4350-5976	tuf-rplC	48.	428-G2c	14047-15468	rpmD-secY
15.	659-648	4438-5465	tuf	49.*	430-G2c	14372-15468	rplO, secY
16.*	732-624c	5240-5976	tuf-rplC	50	634-635	14643-16387	*rplO-adk*
17.*	647-618	5297-5806	tuf, rpsJ	51.*	443-G2c	15276-15468	*secY*
18.*	647-624c	5297-5976	tuf-rplC	52.*	443-G1	15276-15752	*secY*
19.	624-650	5976-6790	rplC, rplD	53.**	SecYII-SecYIV	15289-15946	*secY*
20.*	624-644	5976-7151	rplC-rplW	54.	G2-G1	15468-15752	*secY*
21.	624-621c	5976-7847	rplC-rplB	55.*	G2-444	15468-15970	*secY*
22.*	651-644	6883-7151	rplD, rplW	56.	G2-429	15468-16353	*secY-adK*
23.*	643-621c	7138-7847	rplW, rplB	57.*	G2-400	15468-16640	*secY-infA*
24.*	622-621c	7689-7847	rplB	58.*	260-458c	16616-18104	*infA-rpsD*
25.	621-625	7847-8504	rplB, rpsS	59.*	458-507	18104-18696	*rpsD, rpoA*
26.*	621-605c	7847-9082	rplB-rpsC	60.*	458-504	18104-19376	*rpsD, rpoA*
27.*	605-460	9082-10196	rpsC-rpmC	61.	450-479	18163-19264	*rpsD, rpoA*
28.*	801-803c	10105-10965	rpmC-rplX	62.*	477-504	18584-19376	*rpoA*
29.	801-301c	10105-11601	rpmC-rplE	63.	477-501c	18584-19791	*rpoA, rplQ*
30.	801-300	10105-12110	rpmC-rpsH	64.	503-480	18862-19621	*rpoA, rplQ*
31.*	310-309	10167-10672	rpmC-rplN	65.*	478-501c	19371-19791	*rpoA, rplQ*
32.*	310-277	10167-11107	rpmC-rplX	66.*	478-502	19371-20341	*rpoA, rplQ,*
33.*	310-301c	10167-11601	rpmC-rplE	67.	501-502	19791-20341	*rplQ*
34.*	301-300	11601-12110	rplE-rpsH				

* Fragments used in the phylogenetic analysis from the binary data.

** Primer pair used to produced G1–G2 sequences from all pathogenic species.

### Comparative PCR analysis of the S10-spc-α locus in pathogenic Leptospira

Amplification patterns of different *L. borgpetersenii* and *L. kirschneri* strains shared a high level of identity (one and two differences, respectively). However, marked strain differences were found within the species *L. santarosai* (8), *L. noguchii* (9), *L. weilii* (15) and *L. alexanderi* (14). Predictably, because genetic relatedness is used to differentiate *Leptospira* species, the amplification profiles varied depending on the species from which the template DNA was isolated (Table S3). These data show that strains composing these species likely have higher sequence variation within the *S10-spc-α* locus than that seen in *L. interrogans*. To confirm that failed PCR amplifications were due to sequence variation at or near the primer annealing sites, and not a disruption of gene synteny, a series of additional primers were designed that directed amplification from conserved sequences in adjacent genes through the regions in question. Amplification using these additional primer sets confirmed that all genes initially identified in the *L. interrogans S10-spc-α* locus were present throughout the same locus of all pathogenic *Leptospira* species. This conserved organization extends as far as *fus*, encoding EF-G at the 5′ end of the locus, through *rpsD* at the 3′ end of the locus. Thus the genetic organization of the *S10-spc-α* locus is conserved in all pathogenic *Leptospira* spp. with no signs of disruptions or translocations of genes within the locus.

### Comparative PCR analysis of the S10-spc-α locus of non-pathogenic Leptospira

Attempts to perform PCR analysis of DNA from *Leptospira* species with saprophytic or intermediate (i.e. questionable) pathogenic status frequently failed to generate products or yielded anomalous sized amplicons. These data imply a marked divergence in the *S10-spc-α* sequence content from pathogenic *Leptospira* (Table S3). Interestingly, the amplification profile of *L. meyeri* strain ICF is consistent with a pathogenic status whereas the profile of *L. meyeri* strain Veldrat Semarang 173 is more similar to those of the saprophytic and intermediate species *L. biflexa*, *L. fainei*, and *L. inadai*.

To determine if the genetic composition of this segment of the genome is different between saprophytic and pathogenic *Leptospira*, the corresponding regions of the *L. biflexa*, *L. interrogans*, and *L. borgpetersenii* genomes (GenBank accession numbers for *L. interrogans*, *L. borgpetersenii* and *L. biflexa* are AE016823, CP000348, CP000786, respectively) were aligned with BLAST and the results visualized by ACT (Fig. S1). These data show that saprophytic and pathogenic *Leptospira* have the same organization in the *S10-spc-α* locus, and the lack of successful PCR amplification is likely due to extensive sequence drift within the genus.

### Phylogenetic analysis from binary data

**Figure 1:**
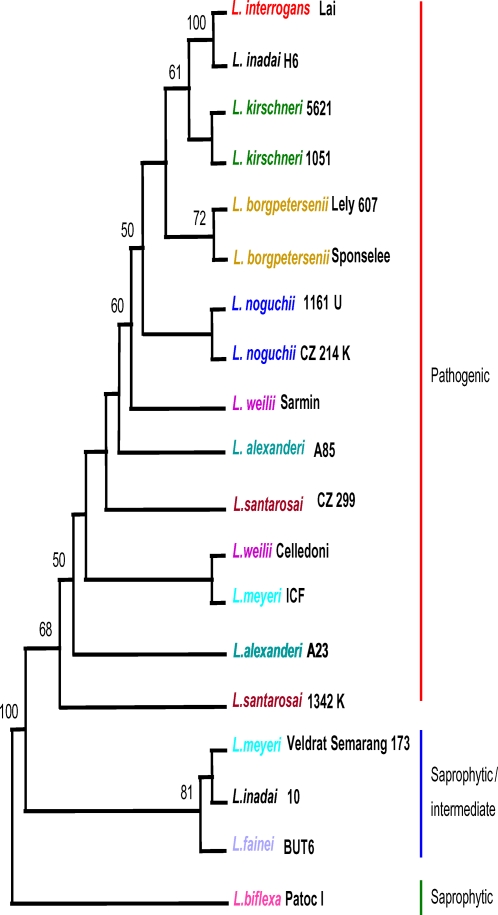
Consensus tree based on PCR amplification data. Majority-rule consensus tree elaborated under the parsimony criterion and based on binary data (absence/presence) coded from amplification patterns in the *S10-spc-α* locus for different *Leptospira* species. Numbers on nodes are bootstrap support after 100 replicates. Only bootstrap values above or equal to 50% are shown. Species included in the sequence analysis are coded in color. *L. biflexa* was used as the outgroup. CI = 0.346.

The parsimony criterion was used to infer phylogenetic relationships within *Leptospira* from binary data. The most parsimonious tree generated from these data shows two prominent well-supported clades: 1) a basal clade, with bootstrapping value of 81%, that includes *L. fainei* and *L. inadai*, two species with intermediate pathogenic status, and the saprophytic *L. meyeri* strain Veldrat Semarang 173; and 2) a sister clade, supported with a 68% bootstrap value, that contains pathogenic *Leptospira* species (Fig. 1). Within the pathogenic clade, relationships among *L. alexanderi*, *L. santarosai*, and *L. weilii* species, are poorly resolved. In contrast, *L. interrogans*, *L. kirschneri*, *L. borgpetersenii*, and *L. noguchii* are clustered in a well-supported clade where *L. kirschneri* and *L. interrogans* appear as closely related siblings.

Surprisingly, there are two exceptions to the predicted distribution of strains. *L. inadai* strain H6 clusters with *L. interrogans*, and *L. meyeri* strain ICF branches within pathogenic species suggesting a pathogenic status for these two strains. As we note in the Discussion section, we believe strain H6 is incorrectly classified as *L. inadai*.

### Multilocus sequence typing (MLST)

Phylogenetic analysis was done on four conserved loci within the *S10-spc-α* locus and compared to available *Leptospira rrs* sequence data (GenBank accession numbers EU365895-EU365966). DNA amplification of target sequences from the intermediate strains *L. fainei* strain BUT 6, *L. inadai* strain 10, and *L. meyeri* strain Veldrat Semarang 173 was not successful. Therefore, these strains were not included in the analysis. The sequences for the saprophytic strain Patoc I were deduced from its genome sequence [17]. None of the analyzed sequences are significantly deviated from neutral expectations (*P*>0.1). The shortest G1–G2 fragment (245 bp) showed the highest nucleotide diversity, π value of 0.14, whereas in the 300–301 fragment π was 0.09. Congruently, the mean divergence values (D) for pairwise comparisons ranged from 0.103 to 0.171 for the 300–301 and G1–G2 fragment, respectively. The lowest phylogenetic signal was obtained for 300–301 sequences. In contrast, the 621–625 and 624–650 fragments showed a phylogenetic signal slightly higher than the combined data set (Table 2).

**Table 2:** Nucleotide diversity, divergent estimations and parameters estimated from the sequences of 4 fragments in diverse *Leptospira* species.

Fragment	Sites	Polymorphic Sites	Mean D	π	Θ per site	Tajima's D	*P*	*-g1*
300–301	469	179	0.1030	0.0891	0.1011	−1.2016	>0.1	−0.5670
621–625	479	176	0.1190	0.1034	0.1306	−0.8672	>0.1	−0.9404
624–650	491	226	0.1440	0.1205	0.1623	−1.0739	>0.1	−1.0876
G1–G2	245	91	0.1710	0.1434	0.1381	0.1606	>0.1	−0.5914

Distance and parsimony analysis yielded identical or similar topologies and bootstrapping values were comparable for the concordant nodes, although they were generally lower in parsimony trees. Alternative branching patterns in parsimony trees (with bootstrap value<50%) occurred in nodes showing the lowest bootstrap support in distance topologies.

**Figure 2:**
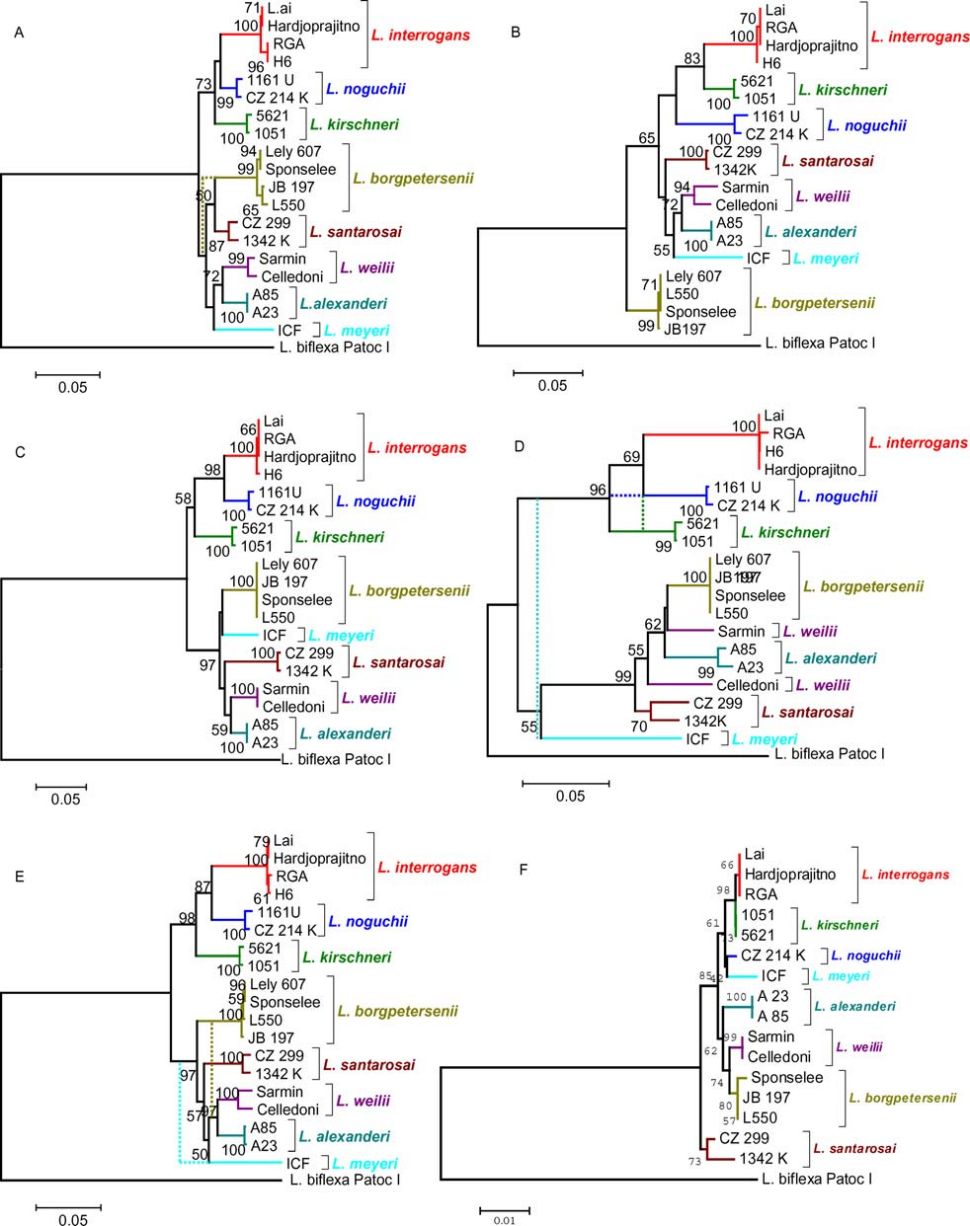
Phylogenetic trees based on Tamura-Nei distances and elaborated using the Neighbor-Joining method. Distances were calculated from the 300–301 (A), 621–625 (B), 624–650 (C) and G1–G2 (D) sequence fragments within the *S10-spc-α* locus of pathogenic species of *Leptospira*. The total evidence was combined and analyzed under identical conditions (E). In addition, data available from 16S rDNA (*rrs*) sequences were used to obtain an alternative hypothesis for the relationships of diverse *Leptospira* strains (F). Dotted lines show alternative branching patterns, with bootstrapping values ≥50%, obtained in the consensus majority rule tree obtained by parsimony criterion. Numbers above branches represent the percentage of bootstrapping results (2000 replicates). Trees are drawn to scale as indicated by the bar depicted below each tree; bars represent the estimated distance in units of the number of base substitutions per site. The scale the 16S rRNA-based tree is expanded relative to other loci. *L. biflexa* was used as the outgroup.

In the composite tree (Fig. 2E), pathogenic strains were separated into two well-supported clades that are similar, but not identical to clades resolved in the binary tree. One clade consists of the sister sub-clades containing *L. interrogans* and *L. noguchii*, with *L. kirschneri* located in a basal position. This clade is consistently recovered in all topologies (Fig. 2), with the exception of the tree based on the 621–625 fragment (Fig. 2B) and parsimony topology generated from G1–G2 sequences (Fig. 2D), where *L. kirschneri* and *L. noguchii* swap their positions. The close relationships of these species are also apparent through comparative analysis using 16S rDNA sequence data (Fig. 2F) and independent binary data (Fig. 1). The second clade of the composite tree contains *L. borgpetersenii*, *L. santarosai*, *L, alexanderi*, *L. weilii* and *L. meyeri* strain ICF. Although, the branching pattern within this clade has lower support, the sibling relationship between *L. alexanderi* and *L. weilii* is well conserved. The relative positions of *L. borgpetersenii*, *L. meyeri* and *L. santarosai* are uncertain and vary depending on the data set and method of analysis (Fig. 2). In the tree inferred from the G1–G2 locus, the Celledoni and Sarmin strains of *L. weilii* are located in separate clades.

The repeated findings that placed *L. inadai* strain H6 within the *L. interrogans* cluster, suggested that this strain is probably misclassified and belongs to *L. interrogans*. To rule out that an incorrect strain was used in our study, we repeated the sequence analysis with an H6 strain originating from the CDC collection used to establish the current taxonomic description of *Leptospira* [2]. Results with the CDC H6 reference strain were identical with results obtained with our strain excluding an error in our collection.

The *S10-spc-α* locus encodes ribosomal proteins that interact with rRNA, therefore ribosomal protein and rRNA sequences are expected to have parallel phylogenies. Because *rrs* is a well-accepted target for phylogenetic analysis we constructed a phylogenetic tree from available *rrs* sequence data. The *rrs* based phylogenetic tree is similar to the locus-deduced tree, showing close relationships between the species *L. interrogans*, *L. kirschneri* and *L. noguchii*. Both the clade support and genetic divergence among other *Leptospira* species based on *rrs* sequence data was lower than for *S10-spc-α* locus data alone, a finding consistent with a slower rate of sequence drift in rRNA than ribosomal protein genes.

### Phylogeny of secY versus its G1–G2 domain

**Figure 3:**
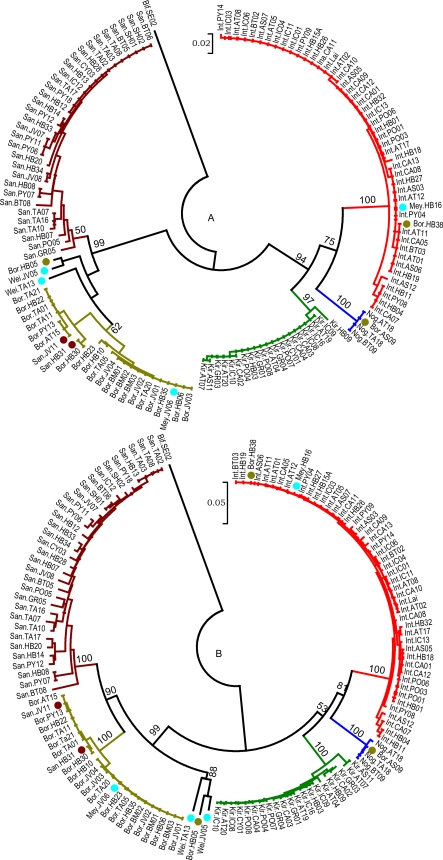
Circular phylogenetic trees based in Tamura-Nei distances and elaborated using Neighbor-Joining method. Distances were calculated from G1–G2 (A) restricted sequences or the *secY* sequences (B), and are based on analysis of 131 strains of pathogenic species of *Leptospira*. Numbers above branches represent the percentage of bootstrapping results (2000 replicates). Only bootstrap values above or equal to 50% are shown. *L. biflexa* was used as the outgroup. Dots indicate strains with divergent positions compared to those from DNA-DNA reassociation analysis [2].

The 20-mer primers G1 and G2 amplify a 285 bp fragment of *secY*, and these primers were developed previously as a diagnostic PCR for the detection of *Leptospira* DNA [5]. A 245 bp fragment flanked by the G1–G2 primers has been shown previously to be a useful tool for discriminating between species [18]–[21]. This study provides an opportunity to broaden the evaluation of the G1–G2 domain by comparing the discriminative value of this domain with the majority of the *secY* sequence. Sequences for *secY* were obtained from 131 *Leptospira* strains (GenBank accession numbers EU357938–EU358070). The phylogenetic tree produced from *secY* sequence data was compared to a tree derived from the extracted sequences of the 245 bp fragment flanked by primers G1 and G2 (Fig. 3). These two trees are similar, resolve *Leptospira* species, and discriminate between strains. With few exceptions, all strains clustered with other members of the same species as determined by DNA-DNA hybridization analysis [4]. Because of a limitation presented by the original G2/G2 primer pair, it does not amplify DNA from *L. kirschneri*; two new primers were designed (SecYII and SecYIV) that flank the G1 and G2 annealing sites. These primers amplify *secY* sequences from all pathogenic strains (data not shown).

## Discussion

Whole genome sequence analyses of different *Leptospira* species reveal extensive plasticity, including rearrangements, duplications, and disruptions of otherwise conserved segments of the genome [10]–[12], [17]. Previously, we demonstrated that *L. interrogans* strain Lai contained a large ribosomal protein locus spanning the *S10*, *spc*, and *α* loci identified in widely divergent eubacterial genera [14]. Notably, this entire locus is transcribed from either of two promoters upstream of *fus*, the first gene in the operon, and comprises one of the longest known prokaryotic transcripts [14]. In the present study, we show that genetic content and organization of the *S10-spc-α* locus is well conserved across the genus *Leptospira*, a finding that is somewhat remarkable given the extent of rearrangements that have disrupted synteny during *Leptospira* evolution. The conserved *S10-spc-α* organization includes the presence of the 5′ *fus* gene coding for elongation factor EF-G and the genes *adk* (adenylate kinase), *infA* (IF1), and *rpsD* (S4) located at the 3′ end of the locus, genes that are dispersed in the *B. burgdorferi* and *T. pallidum* genomes [14]. The genetic organization of the *Leptospira S10-spc-α* locus is unique among spirochetes [14], and the data presented in this work support phylogenetic evidence that suggests *Leptospira* are one of the oldest branches in spirochete evolution. Conservation of the *Leptospira S10-spc-α* locus is in stark contrast to the unique organization of rRNA genes, where the *rrs*, *rrl*, and *rrn* genes are not closely linked to each other, but are dispersed throughout the larger of two chromosomes comprising the *Leptospira* genome [16], [22]. Despite a lack of synteny for the ribosomal RNA genes, rRNA genes show limited sequence divergence. Generally, rRNA sequence conservation is a consequence of low tolerance to change due to structural constraints within the ribosome and a requirement to maintain specific binding sites for ribosomal proteins [23], [24].

PCR analysis of the *S10-spc-α* locus showed a number of regions that were more consistently amplified than other regions (Table S3), suggesting that either this locus has undergone rearrangements or that sequence drift affected the efficiency of primers to faithfully bind template from diverse species. Alignment of genomic sequences spanning the *S10-spc-α* locus showed that the genetic organization of this locus is conserved among pathogenic and saprophytic *Leptospira* (Fig. S1). Therefore, variable success in amplifying regions of the *S10-spc-α* locus from diverse *Leptospira* species is likely due to sequence drift; *Leptospira* species have substantial differences in sequence composition as shown by DNA∶DNA hybridization analysis [2]. Additionally, the PCR primers were designed primarily from the available genomic sequences of two pathogenic *Leptospira* serovars, and our results may be biased due to the divergence between pathogenic and saprophytic species. The binary PCR data positioned *Leptospira* species into two clades; one clade contained only pathogenic species, while the other contained both saprophytic species and species with intermediate pathogenic potential. One important aspect of our findings is confirmation that *L. fainei*, *L. inadai*, and *L. meyeri*, known to present a group of *Leptospira* with intermediate pathogenic potential, form a distinct cluster separate from true pathogenic species, suggesting the presence of three distinct lines of evolution within this genus.

We selected four loci within the *S10-spc-α* locus that were consistently amplified from *Leptospira* species in initial studies to perform phylogeny studies. Phylogenetic trees deduced from the separate loci as well as from the concatenated sequence were similar, and resulted in trees each having two clades, results similar to those obtained from the binary PCR data. The clades contained branches that, with few exceptions, reflected species designations based on *rrs* sequence analysis [25], MLST analysis [26], multilocus enzyme electrophoresis (MLEE) [27], and DNA homology data [2].

Three anomalies were found during comparison of the binary and sequence-based phylogenetic trees. First, the two strains of species *L. meyeri* were separated into different branches. Strain ICF was positioned in the pathogenic clusters whereas Veldrat Semarang 173 appeared in the saprophytic/intermediate pathogen cluster. This is consistent with previous reports that ICF is a pathogenic strain and Veldrat Semarang 173 is a saprophytic one [5], [28]. The findings of this work imply that *L. meyeri* is composed of strains with different pathogenic potential. A second anomaly detected in this work affects the classification of strain H6. Strain H6 was designated a member of *L. inadai* based on DNA∶DNA reassociation analysis [2], but MLEE data contradicted this finding [27]. To exclude the possibility that the discrepancy in previous studies, and in our work, was due to contamination, we analyzed strain H6 from both our collections and the reference collection at CDC used to develop the current species designations using DNA hybridization data [2], and found both strains had identical sequences to *L. interrogans*. Consequently, we recommend that strain H6 be reclassified as *L. interrogans*. The third anomaly involves *L. weilii* strains Celledoni and Sarmin. These two strains are separated into separate clades in the G1–G2 sequence-based tree (Fig. 2E). However, these two strains share the same clade based on analyses using binary data or sequence data. We believe that gene duplication and recombination events might have facilitated horizontal transfer of all or part of *secY* (corresponding to the G1–G2 region). It should be noted that genes contained in the *S10-spc-α* locus are duplicated in *L. borgpetersenii* strain L550, but are found as unique copy genes in all other sequenced *Leptospira* genomes, including *L. borgpetersenii* strain JB197 [10]. Thus, duplication of this locus and subsequent DNA acquisition via horizontal genetic transfer could facilitate stable integration of divergent *secY* genes.

The *S10-spc-α* locus includes the *secY* gene encoding preprotein translocase. Primer pair G1–G2 is positioned within this gene and directs amplification of a 285 bp fragment from all pathogenic species except *L. kirschneri* [5]. Although it has been suggested that this small fragment has a high discriminating power making it useful for a quick speciation [18]–[21], data supporting that contention is fragmentary. One goal of this study was to determine if analysis of the G1–G2 region provided sufficient information for *Leptospira* spp. discrimination. Data generated in the present study is more comprehensive than previous reports; phylogenetic trees based on the G1–G2 segment are in accordance with *rrs* based trees, showing that analysis of this small fragment can be used to identify species.

Genetic analysis of the *S10-spc-α* locus contributes to a better understanding of *Leptospira* evolution. Trees generated from analysis of sequence data generated here provide analysis of more conserved loci than those studied previously [25], and may be more useful in comparing evolution of the genus. A conserved, yet distinct genetic organization of this locus provides additional support for the early divergence of *Leptospira* from other spirochetes. Finally, from a practical standpoint, we demonstrate that analysis of a 245 bp segment of *secY* is suitable for rapid identification of *Leptospira* species.

## Materials and Methods

### Bacterial strains and media

*Leptospira* strains used in this study were from the reference collections of the WHO/FAO/OIE Collaborating Center for Reference and Research on Leptospirosis at KIT Biomedical Research, Amsterdam, The Netherlands, and the USDA Leptospirosis Reference Center at the National Centers for Animal Health, Ames, USA (Table S1). Bacteria were propagated at 30°C in EMJH liquid media as described by Ellinghausen and McCullough [29] as modified by Johnson and Harris [30].

### DNA extraction

*Leptospira* were grown to late log phase, harvested by centrifugation, and genomic DNA was extracted using a QIAamp DNA mini kit (Qiagen, Germany) following the manufacturer's instructions. DNA concentration was determined using a Nano-Drop-1000 spectrophotometer (ThermoFisher Scientific, USA) and by visual comparison with Smart Ladder SF (Eurogentec S.A., Belgium) after agarose gel electrophoresis in 1.5% agarose gels, stained with ethidium bromide according to standard procedures [31].

### PCR analysis

Adjacent and overlapping fragments from the whole *S10-spc-α* locus were amplified by PCR from various *Leptospira* strains using primers listed in Table 1 and S2. Several primers were designed by cross-species alignment of available *L. interrogans* and *L. borgpetersenii* genome sequences [10]–[12] and access to the *L. biflexa* Patoc I genome sequence before publication (D. Bulach [17]). In addition, an iterative approach was used to develop primers useful for sequencing *secY* by identifying conserved regions suitable for amplification of adjacent variable regions across divergent species for which the genome sequences are yet unavailable. Primer sets were designed to produce a series of overlapping amplification products to ensure the presence and correct location of genes in the locus.

PCR amplifications were done on a PTC-100 Peltier Thermal Cycler (MJ Research, USA) using the following program: denaturation for 5 min. at 94°C, followed by 34 cycles consisting of annealing, 1 min at 52°C, primer extension, 2 min at 72°C, denaturation, 1 min at 94°C. PCR products were separated by agarose gel electrophoresis and visualized as described above.

### Sequencing

PCR amplification products were purified by QIAquick PCR purification kits (Qiagen Corp.) prior to DNA sequencing. Nucleotide sequences were determined by dye termination reactions separated on ABI Prism 310 and ABI 3700 (Applied Biosystems, USA) DNA sequencers. Sequencing was done on both complementary and forward strands and repeated at least twice to obtain reliable sequence data. Sequence data were edited using Sequencher (Gene Codes Corp., USA).

### Phylogenetic analysis: Binary Analysis of PCR data

The presence (1) or absence (0) of correctly amplified fragments within the *S10-spc-α* locus, for each of the analyzed species, was codified in a discrete binary 40-character matrix covering a complete set of 24 taxa, representing eleven *Leptospira* species (Table S1). The characters were weighted proportionally to fragment size and assumed sequence homology for fragments with identical estimated size. The data matrix was analyzed under parsimony criteria using the branch and bound algorithm; support for branches in the unrooted tree was estimated by bootstrapping (100 replicates) with the program PAUP* v. 4.0b10 [32]. The inferred phylogenetic relationships are based on both gene organization and sequence variation within the complete *S10-spc-α* locus. Phylogenetic signals contained in this data set were evaluated by *g*_1_ estimation (g_1_ = −0.947). The negative skew of the distribution of three lengths, under parsimony criterion, is originated from trees with low scores based in highly informative data [33].

### Phylogenetic analysis: Comparative Sequence Analysis

Sequence data from four loci within the *S10-spc-α* locus were obtained to conduct a distance and parsimony-based phylogenetic analysis of pathogenic *Leptospira* using MEGA4 and PAUP* v. 4.0b10, respectively. Nucleotide diversity and diverse sequence parameters were obtained with MEGA4 [34] and DNASP [35]. The hypothesis that all mutations are selectively neutral was tested using Tajima's D test [36] implemented in DNASP. The confidence limits of *D* (two-tailed test) was obtained assuming that *D* follows the beta distribution and the confidence limits given in equation 47 and Table 2, respectively in Tajima, 1989 [36]. Confidence intervals were also determined for Tajima's *D* by computer simulations using the coalescent algorithm. In distance analysis, midpoint rooted trees were obtained by the neighbor-joining method with Tamura-Nei distances [37], [38] and the cluster support was estimated by bootstrapping with 2000 replicates [39]. The gaps were ignored only when they are included in the two sequences compared, using the pairwise-deletion option. In parsimony analysis, a branch-and-bound search was used with 2000 bootstraps. The homogeneity of the four partitioned data sets was evaluated using the incongruence-length difference test [40] implemented in PAUP* v. 4.0b10. An initial tree was inferred from a data set that concatenated all the available sequenced fragments, i.e. data from the *rplE*, *rpsN*, *rpsH* (primer pair 301–300), *rplB*, *rpsS* (primer pair 621–625), *rplC*, *rplD* (primer pair 624–650) and *secY* (primer pairs G1–G2) (Table 1). This data set represents 1684 bp from each of nine *Leptospira* species and 19 representative strains, including *L. biflexa* strain Patoc I as an outgroup. Concurrently, sequence data from each of the individual fragments used in the concatenated set were analyzed separately using identical analysis methodology, to search for topological local incongruence responsible for the low support of particular nodes in the initial tree. In addition, two *secY* fragments (spanning the G1–G2 and SecY II–IV primer sets, respectively) were used for the reconstruction of phylogenetic relationships between 131 strains, using *L. biflexa* strain Patoc I as an outgroup. The *secY* sequences were stripped to a standard size of 1289 bp, whereas the sequences derived from G1–G2 were significantly shorter: 245 bp. Using this latter fragment, a comparison of monophyletic clustering and resolution of *Leptospira* species respect to the standard 16S rDNA data was done.

### ACT Alignment

Alignment of *S10-spc-α* locus sequences from *L. interrogans*, *L. borgpetersenii*, and *L. biflexa* was done using BLASTN [41] with settings adjusted to identify regions having ≥80% sequence identity. Data were visualized using the Artemis Comparison Tool (ACT) [42].

## Supporting Information

Figure S1 Alignment of the L. biflexa, L. interrogans and L. borgpetersenii S10-spc-a genome sequences. Regions of greater than 80% sequence identity are shown as blue (between L. interrogans and L. borgpetersenii) and red (L. biflexa and L. interrogans). White regions indicate segments where sequence identity drops below 80%. Regions of similarity were determined using Blastn under default settings except the -m 8 output option was used. The display was generated using ACT. Note that the orientation of these sequences shown in the figure is consistent with the genomic sequence data in GenBank and are inverted relative to the direction of transcription. GenBank accession numbers for the genomes of L. interrogans, L. borgpetersenii and L. biflexa are AE016823, CP000348, CP000786, respectively.
(2.24 MB DOC)

Table S1 Leptospira strains used for the S10-spc-α locus study.
(0.25 MB DOC)

Table S2 All primers used for the S10-spc-α locus analysis.
(0.09 MB DOC)

Table S3 Amplification through the S10-spc-α operon of Leptospira spp. Positive and negative PCR scores for amplification reactions along the locus from various strains.
(0.05 MB DOC)
