# Supplementary figures and images for: Conservation of the S10-spc-α Locus within Otherwise Highly Plastic Genomes Provides Phylogenetic Insight into the Genus Leptospira
Source: PLoS One. 2008 Jul 16;3(7):e2752. doi: 10.1371/journal.pone.0002752 (PMC2481283; doi:10.1371/journal.pone.0002752)

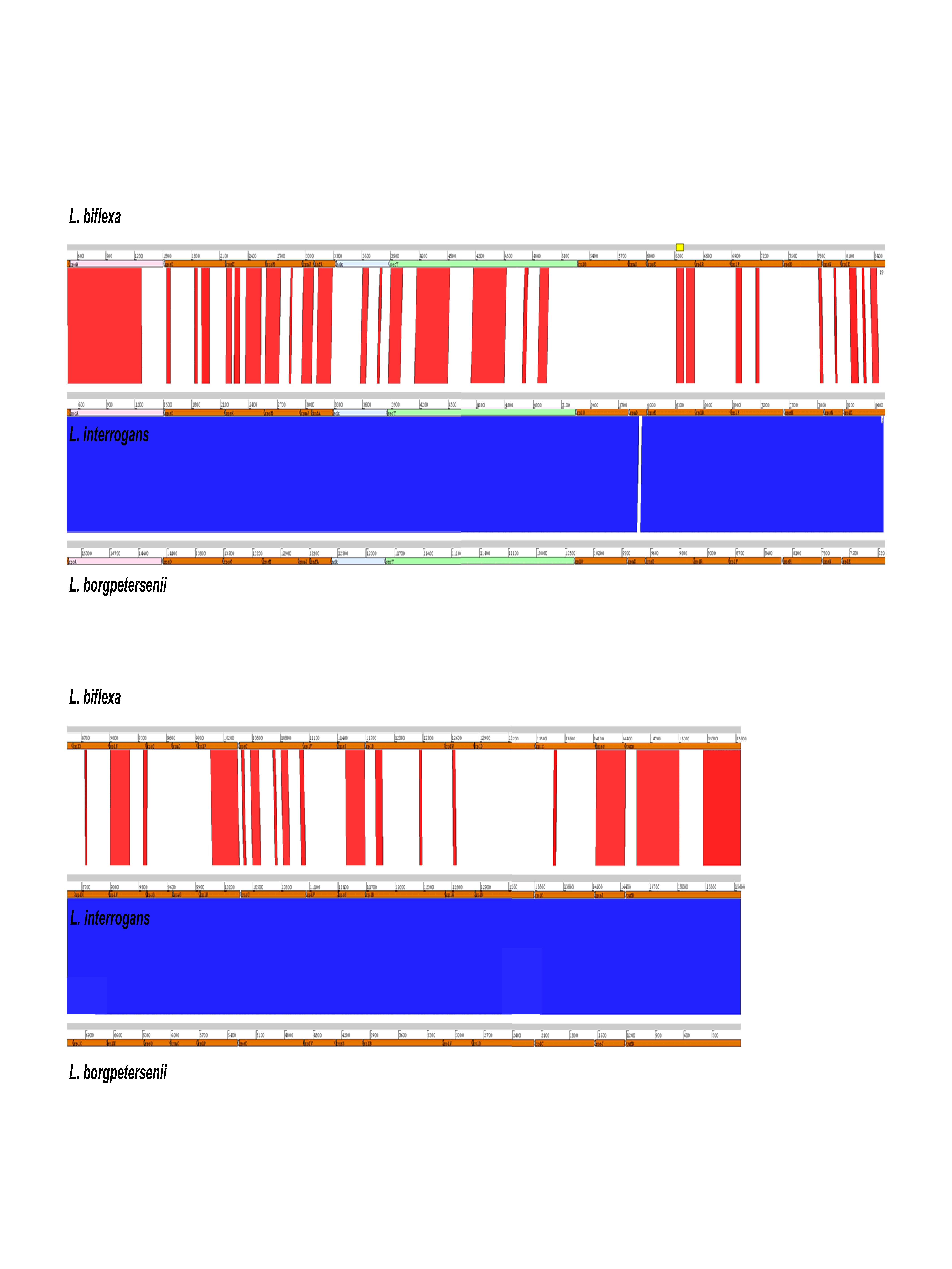

Supplement: Figure S1 — Alignment of the L. biflexa, L. interrogans and L. borgpetersenii S10-spc-a genome sequences. Regions of greater than 80% sequence identity are shown as blue (between L. interrogans and L. borgpetersenii) and red (L. biflexa and L. interrogans). White regions indicate segments where sequence identity drops below 80%. Regions of similarity were determined using Blastn under default settings except the -m 8 output option was used. The display was generated using ACT. Note that the orientation of these sequences shown in the figure is consistent with the genomic sequence data in GenBank and are inverted relative to the direction of transcription. GenBank accession numbers for the genomes of L. interrogans, L. borgpetersenii and L. biflexa are AE016823, CP000348, CP000786, respectively. (2.24 MB DOC) [file pone.0002752.s001.tif]
