# Supplementary material for: Conservation of the S10-spc-α Locus within Otherwise Highly Plastic Genomes Provides Phylogenetic Insight into the Genus Leptospira
Source: PLoS One. 2008 Jul 16;3(7):e2752. doi: 10.1371/journal.pone.0002752 (PMC2481283; doi:10.1371/journal.pone.0002752)
Supplement: Table S1 — Leptospira strains used for the S10-spc-α locus study. (0.25 MB DOC) [file pone.0002752.s002.doc]

**Table S1.** *Leptospira* strains used for the *S10*-*spc*-α locus study.

| **No** | **Code** | **Serovar** | **Serogroup** | **Strain** | **Species** | **Ref.^#^** |
| --- | --- | --- | --- | --- | --- | --- |
| 1. *+ | Alex.A23 | Manzhuang | Hebdomadis | A23 | *L. alexanderi* | A |
| 2. *+ | Alex.A85 | Mengla | Javanica | A85 | *L. alexanderi* | A |
| 3. *+ | Bif.SE02 | Patoc | Semaranga | Patoc I | *L. biflexa* | A |
| 4. | Bor.HB22 | Balcanica | Sejroe | 1627 Burgas | *L. borgpetersenii* | A |
| 5. | Bor.BM01 | Ballum | Ballum | Mus 127 | *L. borgpetersenii* | A |
| 6. | Bor.BM02 | Ballum | Ballum | S102 | *L. borgpetersenii* | A |
| 7. | Bor.BM03 | Castellonis | Ballum | Castellon 3 | *L. borgpetersenii* | A |
| 8. | Bor.JV02 | Ceylonica | Javanica | Piyasena | *L. borgpetersenii* | A |
| 9. | Bor.TA20 | Dikkeni | Sejroe | Mannuthi | *L. borgpetersenii* | A |
| 10. * | Bor.JB197 | Hardjo-bovis | Sejroe | JB197 | *L. borgpetersenii* | C |
| 11. *+ | Bor.Lely607 | Hardjo-bovis | Sejroe | Lely 607 | *L. borgpetersenii* | C |
| 12. * | Bor.L550 | Hardjo-bovis | Sejroe | L550 | *L. borgpetersenii* | C |
| 13. *+ | Bor.Sponselee | Hardjo-bovis | Sejroe | Sponselee | *L. borgpetersenii* | A |
| 14. | Bor.JV01 | Javanica | Javanica | Veldrat Batavia 46 | *L. borgpetersenii* | A |
| 15. | Bor.HB06 | Jules | Hebdomadis | Jules | *L. borgpetersenii* | A |
| 16. | Bor.TA05 | Kisuba | Tarassovi | Kisuba | *L. borgpetersenii* | A |
| 17. | Bor.PY13 | Kwale | Pyrogenes | Julu | *L. borgpetersenii* | A |
| 18. | Bor.HB10 | Mini | Mini | Sari | *L. borgpetersenii* | A |
| 19. | Bor.TA21 | Moldaviae | Bataviae | 114-2 | *L. borgpetersenii* | A |
| 20. | Bor.HB35 | Nero | Sejroe | Gamsulin | *L. borgpetersenii* | A |
| 21. | Bor.HB30 | Nyanza | Sejroe | Kibos | *L. borgpetersenii* | A |
| 22. | Bor.AS09 | Pina | Australis | LT 932 | *L. borgpetersenii* | A |
| 23. | Bor.JV03 | Poi | Javanica | Poi | *L. borgpetersenii* | A |
| 24. | Bor.HB23 | Polonica | Sejroe | 493 Poland | L. borgpetersenii | A |
| 25. | Bor.JV04 | Sorexjalna | Javanica | Sorex Jalna | L. borgpetersenii | A |
| 26. | Bor.AT15 | Srebarna | Autumnalis | 1409/69 | L. borgpetersenii | A |
| 27. | Bor.TA01 | Tarassovi | Tarassovi | Perepelitsin | L. borgpetersenii | A |
| 28. | Bor.TA11 | Tunis | Tarassovi | P 2/65 | L. borgpetersenii | A |
| 29. | Bor.HB05 | Worsfoldi | Hebdomadis | Worsfold | *L. borgpetersenii* | A |
| 30. + | Fain.BUT 6 | Hurstbridge | Hurstbridge | BUT 6 | *L. fainei* | B |
| 31. + | Ina.10 | Lyme | Lyme | 10 | *L. inadai* | A |
| 32. *+● | Ina.CA11 | Malaya | Canicola | H 6 | *L. inadai* | A |
| 33. | Int.AT01 | Autumnalis | Autumnalis | Akiyami A | *L. interrogans* | A |
| 34. | Int.AS07 | Bangkok | Australis | Bangkok D-92 | *L. interrogans* | A |
| 35. | Int.BT02 | Bataviae | Bataviae | Van Tienen | *L. interrogans* | A |
| 36. | Int.CA10 | Benjamini | Canicola | Benjamin | *L. interrogans* | A |
| 37. | Int.CA08 | Bindjei | Canicola | Bindjei | *L. interrogans* | A |
| 38. | Int.PY04 | Biggis | Pyrogenes | Biggs | *L. interrogans* | A |
| 39. | Int.IC06 | Birkini | Icterohaemorrhagiae | Birkin | *L. interrogans* | A |
| 40. | Int.AS05 | Bratislava | Australis | Jez Bratislava | *L. interrogans* | A |
| 41. | Int.CA07 | Broomi | Canicola | Patane | *L. interrogans* | A |
| 42. | Int.IC11 | Budapest | Icterohaemorrhagiae | PV 1 | *L. interrogans* | A |
| 43. | Int.PY14 | Camlo | Pyrogenes | LT 64-67 | *L. interrogans* | A |
| 44.  + | Int.CA01 | Canicola | Canicola | Hond Utrecht IV | *L. interrogans* | A |
| 45.  + | Int.M20 | Copenhageni | Icterohaemorrhagiae | M20 | *L. interrogans* | A |
| 46. | Int.AT11 | Djasiman | Autumnalis | Djasiman | *L. interrogans* | A |
| 47. | Int.AS06 | Fugis | Australis | Fudge | *L. interrogans* | A |
| 48. | Int.IC13 | Gem | Icterohaemorrhagiae | Simon | *L. interrogans* | A |
| 49. | Int.HB32 | Geyaweera | Sejroe | Geyaweera | *L. interrogans* | A |
| 50. | Int.At12 | Gurungi | Djasiman | Gurung | *L. interrogans* | A |
| 51. | Int.HB26 | Haemolytica | Sejroe | Marsh | *L. interrogans* | A |
| 52. *+ | Int.HB15A | Hardjo | Sejroe | Hardjoprajitno | *L. interrogans* | A |
| 53. | Int.AS12 | Hawain | Australis | LT 62-68 | *L. interrogans* | A |
| 54. | Int.HB01 | Hebdomadis | Hebdomadis | Hebdomadis | *L. interrogans* | A |
| 55. *+ | Int.IC01 | Icterohaemorrhagiae | Icterohaemorrhagiae | RGA | *L. interrogans* | A |
| 56. | Int.CA05 | Jonsis | Canicola | Jones | *L. interrogans* | A |
| 57. | Int.PO06 | Kennewicki | Pomona | LT 1026 | *L. interrogans* | A |
| 58. | Int.HB04 | Kremastos | Hebdomadis | Kremastos | *L. interrogans* | A |
| 59. | Int.CA13 | Kuwait | Canicola | 136/2/2 | *L. interrogans* | A |
| 60. *+ | Int.Lai | Lai | Icterohaemorrhagiae | Lai | *L. interrogans* | A |
| 61. | Int.PY09 | Manilae | Pyrogenes | LT 398 | *L. interrogans* | A |
| 62. | Int.IC03 | Mankarso | Icterohaemorrhagiae | Mankarso | *L. interrogans* | A |
| 63. | Int.HB18 | Medanensis | Sejroe | Hond HC | *L. interrogans* | A |
| 64. | Int.PO03 | Monjakov | Pomona | Monjakov | *L. interrogans* | A |
| 65. | Int.AT05 | Mooris | Autumnalis | Moores | *L. interrogans* | A |
| 66. | Int.AS03 | Muenchen | Australis | München C 90 | *L. interrogans* | A |
| 67. | Int.IC04 | Naam | Icterohaemorrhagiae | Naam | *L. interrogans* | A |
| 68. | Int.BT03 | Paidjan | Bataviae | Paidjan | *L. interrogans* | A |
| 69. + | Int.PO01 | Pomona | Pomona | Pomona | *L. interrogans* | A |
| 70. | Int.CA12 | Portlandvere | Canicola | My 1039 | *L. interrogans* | A |
| 71. | Int.AT02 | Rachmati | Autumnalis | Rachmat | *L. interrogans* | A |
| 72. | Int.HB27 | Ricardi | Sejroe | Richardson | *L. interrogans* | A |
| 73. | Int.PY08 | Robinsoni | Pyrogenes | Robinson | *L. interrogans* | A |
| 74. | Bor.HB38 | Roumanica | Sejroe | TM 294 | *L. interrogans* | A |
| 75. | Int.CA09 | Schueffneri | Canicola | Vleermuis 90 C | *L. interrogans* | A |
| 76. | Int.AT08 | Sentot | Djasiman | Sentot | *L. interrogans* | A |
| 77. | Int.HB11 | Szwajizak | Mini | Szwajizak | *L. interrogans* | A |
| 78. | Int.AT17 | Weerasinghe | Autumnalis | Weerasinghe | *L. interrogans* | A |
| 79. | Int.HB19 | Wolffi | Sejroe | 3705 | *L. interrogans* | A |
| 80. | Kir.CA03 | Bafani | Canicola | Bafani | *L. kirschneri* | A |
| 81. *+ | Kir.AT20 | Bim | Autumnalis | 1051 | *L. kirschneri* | A |
| 82. | Kir.IC16 | Bogvere | Icterohaemorrhagiae | LT 60-69 | *L. kirschneri* | A |
| 83. | Kir.AT07 | Bulgarica | Autumnalis | Nicolaevo | *L. kirschneri* | A |
| 84. | Kir.AT19 | Butembo | Autumnalis | Butembo | *L. kirschneri* | A |
| 85. | Kir.CY01 | Cynopteri | Cynopteri | 3522 C | *L. kirschneri* | A |
| 86. | Kir.AT04 | Erinaceiauriti | Autumnalis | Erineceus Auritus 670 | *L. kirschneri* | A |
| 104. | Mey.JV06 | Sofia | Javanica | Sofia 874 | *L. meyeri* | A |
| 105. | Nog.BT09 | Argentiniensis | Bataviae | Peludo | *L. noguchii* | A |
| 106. | Nog.TA18 | Carimagua | Shermani | 9160 | *L. noguchii* | A |
| 107. | Nog.AT18 | Huallaga | Djasiman | M 7 | *L. noguchii* | A |
| 108. *+ | Nog.CZ214K | Panama | Panama | CZ 214 K | *L. noguchii* | A |
| 109. *+ | Nog.1161U | Proechimys | Pomona | 1161 U | *L. noguchii* | A |
| 110. | San.PY07 | Alexi | Pyrogenes | HS-616 | *L. santarosai* | A |
| 111. | San.TA08 | Atchafalaya | Tarassovi | LSU 1013 | *L. santarosai* | A |
| 112. | San.TA03 | Atlantae | Tarassovi | LT 81 | *L. santarosai* | A |
| 113. | San.SH02 | Babudieri | Shermani | CI 40 | *L. santarosai* | A |
| 114. | San.PY06 | Bagua | Pyrogenes | MW-12 | *L. santarosai* | A |
| 115. | San.TA02 | Bakeri | Tarassovi | LT 79 | *L. santarosai* | A |
| 116. | San.BT06 | Balboa | Bataviae | 735 U | *L. santarosai* | A |
| 117. | San.HB12 | Beye | Mini | 1537 U | *L. santarosai* | A |
| 118. | San.HB08 | Borincana | Hebdomadis | HS 622 | *L. santarosai* | A |
| 119. | San.BT08 | Brasiliensis | Bataviae | An 776 | *L. santarosai* | A |
| 120. | San.GR05 | Canalzonae | Grippotyphosa | CZ 188 | *L. santarosai* | A |
| 121. | San.PY18 | Cenepa | Pyrogenes | MW-2 | *L. santarosai* | A |
| 122. | San.TA16 | Darien | Tarassovi | 637 K | *L. santarosai* | A |
| 123. | San.JV11 | Fluminense | Javanica | Aa 3 | *L. santarosai* | A |
| 124. | San.TA07 | Gatuni | Tarassovi | 1473 K | *L. santarosai* | A |
| 125. | San.HB13 | Georgia | Mini | LT 117 | *L. santarosai* | A |
| 126. | San.HB34 | Goiano | Hebdomadis | Bovino 131 | *L. santarosai* | A |
| 127. | San.HB28 | Gorgas | Sejroe | 1413 U | *L. santarosai* | A |
| 128. | San.HB33 | Guaricura | Sejroe | Bov. G | *L. santarosai* | A |
| 129. | San.BT05 | Kobbe | Bataviae | CZ 320 | *L. santarosai* | A |
| 130. | San.HB07 | Maru | Hebdomadis | CZ 285 | *L. santarosai* | A |
| 131. | San.CY03 | Naparuca | Cynopteri | NN-1 | *L. santarosai* | A |
| 132. | San.TA17 | Navet | Tarassovi | TRVL 109873 | *L. santarosai* | A |
| 133. | San.PY12 | Princestown | Pyrogenes | TRVL 112499 | *L. santarosai* | A |
| 134. | San.TA10 | Rama | Tarassovi | 316 | *L. santarosai* | A |
| 135. | San.JV08 | Rio | Sarmin | Rr 5 | *L. santarosai* | A |
| 136. | San.HB31 | Ruparupae | Mini | M 3 | *L. santarosai* | A |
| 137. | San.PY11 | Sanmartini | Pyrogenes | CT 63 | *L. santarosai* | A |
| 138. *+ | San.SH01 | Shermani | Shermani | 1342 K | *L. santarosai* | A |
| 139.  ! | San.HB14 | Tabaquite | Mini | TRVL 3214 | *L. santarosai* | A |
| 140. | San.HB20 | Trinidad | Sejroe | TRVL 34056 | *L. santarosai* | A |
| 141. *+ | San.PO05 | Tropica | Pomona | CZ 299 | *L. santarosai* | A |
| 142. | San.JV07 | Vargonicas | Javanica | 24 | *L. santarosai* | A |
| 143. | San.IC12 | Weaveri | Sarmin | CZ 390 | *L. santarosai* | A |
| 144. *+ | Wei.Celledoni | Celledoni | Celledoni | Celledoni | *L. weilii* | A |
| 145. | Wei.JV05 | Coxi | Javanica | Cox | *L. weilii* | A |
| 146. | Wei.TA13 | Langati | Tarassovi | M39039 | *L. weilii* | A |
| 147. *+ | Wei.Sarmin | Sarmin | Sarmin | Sarmin | *L. weilii* | A |

* Strains used in MLST analysis, + Strains used in the binary data analysis, ● Strain reclassified as *L. interrogans* from this paper,

! Mislabeled by the reference.

^#^ References: (A) is Brenner et al., 1999; (B) is Perolat et al., 1998; (C) is ‘This paper’.
