# Supplementary material for: Conservation of the S10-spc-α Locus within Otherwise Highly Plastic Genomes Provides Phylogenetic Insight into the Genus Leptospira
Source: PLoS One. 2008 Jul 16;3(7):e2752. doi: 10.1371/journal.pone.0002752 (PMC2481283; doi:10.1371/journal.pone.0002752)
Supplement: Table S2 — All primers used for the S10-spc-α locus analysis. (0.09 MB DOC) [file pone.0002752.s003.doc]

**Table S2. All the primers used for the *S10-spc-α* locus analysis.**

| **Primer** | **Sequence** | **Primer** | **Sequence** |
| --- | --- | --- | --- |
| 191^R^ | 5′ AACCTGACCGACAAGTTGTT 3′ | 740 ^F^ | 5′ CCAGAAGTTGTCCCAAGTA 3′ |
| 258 ^R^ | 5′ GTTTTCTTGCTTCACTTC 3′ | 743^R^ | 5′ TGAGTCCGTGAGGTTTCT 3′ |
| 260 ^F^ | 5′ ATGGCGTTGGAAGTCTCG 3′ | 744^R^ | 5′ TTTCTCGACCAGAAACTC 3′ |
| 277 ^R^ | 5′ TTCTACAATGACTCGGTCTCT 3′ | 745^R^ | 5′ TCCGTAATTGACATTCTT 3′ |
| 300 ^R^ | 5′ CGATTGGATCTGACATACTC 3′ | 748 ^F^ | 5′ AATTATTACATCACGGAATC 3′ |
| 301 ^F^ | 5′ GGTCACTTTGCGCGGAGACT 3′ | 751^R^ | 5′ TCGGTTGTCCCTTCTTCTA 3′ |
| 309 ^R^ | 5′ ATCTCCCACGGAAGCGTAA 3′ | 752 ^F^ | 5′ AGGATTGGGATAAACATTGA 3′ |
| 310 ^F^ | 5′ CGCGAAACTTCTTACTATT 3′ | 800 ^F^ | 5′ ATTCGGAATTGGTAATTCTC 3′ |
| 314 ^F^ | 5′ GTTTCTACTCCAGGAAGAC 3′ | 801 ^F^ | 5′ TCTGGTTCTTTGACCATTTA 3′ |
| 400 ^R^ | 5′ TGCCGTCAACTGTAATCG 3′ | 802 ^F^ | 5′ ACCGCCTCCTTCAACAATAC 3′ |
| 428 ^F^ | 5′ TTAAGAAGAACCGGACAACA 3′ | 2260^F^ | 5′ CGAAGGACTTCTCGGAATGGTGGATC 3′ |
| 429 ^R^ | 5′ TCGGAAATCCGTCCAACAAA 3′ | 2261^R^ | 5′ CCCGTAGAAATCTGAGGGATAGAAAG 3′ |
| 430 ^F^ | 5′ GGTTCGAGGGCGGCCAGA 3′ | 2262^F^ | 5′ GTACGGAACCGTCATTCTTTGTGCG 3′ |
| 443 ^F^ | 5′ GGAATTGGTAACGGAATTTCT 3′ | 2263^F^ | 5′ CGGAAGAAAAATGGTTCAGGCGAAAAGC 3′ |
| 444 ^R^ | 5′ AAGAGAAGTTCCACCGAATG 3′ | 2264^F^ | 5′ ACGATCATTCAGTGGTTGTCTTC 3′ |
| 450 ^F^ | 5′ GAAACTTTATTGCTCATAGAC 3′ | 2265^R^ | 5′ CTTTCTTCCTTCTTCCCCTTCTTTTTG 3′ |
| 458 ^F^ | 5′ AAGAAGATTGGATAATGTAG 3′ | 2266^R^ | 5′ ACCATAGATTCAGGAAGTCTTCCGATG 3′ |
| 460 ^R^ | 5′ AGGATTAGCCTTCAATTGTC 3′ | 2267^R^ | 5′ TCGCTACTGGAAGACAACCACTGAATG 3′ |
| 477 ^F^ | 5′ CGACAACTATCGGTAACT 3′ | 2313^R^ | 5′ CCTGAGTTGGAACTCAAATCTAAG 3′ |
| 478 ^F^ | 5′ CGATCAGTGTCTCCAAGAGT 3′ | 2314^R^ | 5′ TTCCGTACTGCAGAGGAACCTTTC 3′ |
| 479 ^R^ | 5′ AGAACGAACGGAAAGCTC 3′ | 2315^R^ | 5′ GGAGGTCCCATGAAGATAATGTTC 3′ |
| 480 ^R^ | 5′ GGCTTCAAATCGGTAACA 3′ | 2316^R^ | 5′ ACTGCCCATCCAGCCCATTCTTGAC 3′ |
| 501 ^F^ | 5′ ATTCTGAAGTCGGAATTT 3′ | 2317^R^ | 5′ TCCTCTTTCGGTGATCTGTTCC 3′ |
| 502 ^R^ | 5′ AACTCAGCGAATGCACTT 3′ | 2321^R^ | 5′ TCGGTGATCTGTTCTCCTAACC 3′ |
| 503 ^F^ | 5′ CCGGATCTTCATATCGCTAC 3′ | 2322^F^ | 5′ CTGCCTGAGTCTATGGTTCAAC 3′ |
| 504 ^R^ | 5′ TTCAACTCTTGGAGACACT 3′ | 2326^R^ | 5′ TCGATTGGCTTTTTGCTTGAACCATC 3′ |
| 507 ^R^ | 5′ CGTTACGTCCTCTGCAACT 3′ | 2327^R^ | 5′ ATTACAGAAGCATTGATTAAACCTGGG 3′ |
| 605 ^F^ | 5′ CCTGAGAAGATCAATGTA 3′ | 2328^F^ | 5′ TCCTTAACCGTTCTTTTGACTCAGG 3′ |
| 618 ^R^ | 5′ GGATTTGATATCTACGGATACTC 3′ | 2329^R^ | 5′ GAACCATTTTTCTTCCGACCATTTG 3′ |
| 621 ^F^ | 5′ AAACGTGCATAACGTGGAAC 3′ | 2330^F^ | 5′ CAAATGGTCGGAAGAAAAATGGTTCAG 3′ |
| 622 ^F^ | 5′ CGTTCCGCGTTCATTTCT 3′ | 191c ^F^ | 5′ AACAACTTGTCGGTCAGGTT 3′ |
| 624 ^F^ | 5′ GATACGAAGCGATTCAATTA 3′ | 301c ^R^ | 5′ AGTCTCCGCGCAAAGTGACC 3′ |
| 625 ^R^ | 5′ ACGGAAAGTTCTTGTAGG 3′ | 428c ^R^ | 5′ TGTTGTCCGGTTCTTCTTAA 3′ |
| 634 ^F^ | 5′ GGGCGGATCTATCAAATTAC 3′ | 430c ^R^ | 5′ TCTGGCCGCCCTCGAACC 3′ |
| 635 ^R^ | 5′ AAGTGTATCTAAAGCTTCCGC 3′ | 458c ^R^ | 5′ CTACATTATCCAATCTTCTT 3′ |
| 643 ^F^ | 5′ CACCCATAGTTACTGAGAA 3′ | 501c ^R^ | 5′ AAATTCCGACTTCAGAAT 3′ |
| 644 ^R^ | 5′ AGATCCTGGGATTTCTCA 3′ | 605c ^R^ | 5′ TACATTGATCTTCTCAGG 3′ |
| 647 ^F^ | 5′ ATAACTACCGTCCTCAGTTT 3′ | 621c ^R^ | 5′ GTTCCACGTTATGCACGTTT 3′ |
| 648 ^R^ | 5′ CGGATCCAATGGTTCTTC 3′ | 624c ^R^ | 5′ TAATTGAATCGCTTCGTATC 3′ |
| 651 ^F^ | 5′ ACGAAGGCATTTGATTCT 3′ | 743c ^F^ | 5′ AGAAACCTCACGGACTCA 3′ |
| 657 ^F^ | 5′ AACAATTGGTCACGTAGA 3′ | 803c ^R^ | 5′ CCTCCGGAGCCAGAGAGATAA 3′ |
| 659 ^F^ | 5′ GCCGTTGCTTATGACCAA 3′ | G1 ^R^ | 5′ CTGAATCGCTGTATAAAAGT 3′ |
| 660 ^R^ | 5′ CGTTTAAGTGAGGTTTAGA 3′ | G2 ^F^ | 5′ GGAAAACAAATGGTCGGAAG 3′ |
| 667 ^R^ | 5′ TTTGCAAGTGTAGTAGTAATTG 3′ | G2c ^R^ | 5′ CTTCCGACCATTTGTTTTCC 3′ |
| 706 ^R^ | 5′ AATGCTGATGTAATGGCTTCT 3′ | R1 ^F^ | 5′ GAAGCAGGGCTGGAGTTC 3′ |
| 729 ^R^ | 5′ TTCCTAAGACCGCTTCTA 3′ | R1c ^R^ | 5′ GAACTCCAGCCCTGCTTC 3′ |
| 732 ^F^ | 5′ CCGCTGAGGTGTATGTAT 3′ | SeqY II ^F^ | 5′ GAATTTCTCTTTTGATCTTCG 3′ |
| 735 ^F^ | 5′ GAATCTTTGCCCACATAGA 3′ | SeqY IV ^R^ | 5′ GAGTTAGAGCTCAAATCTAAG 3′ |
| 737 ^F^ | 5′ CAGTCCGTGGTGGAAGAT 3′ |  |  |

F sense

R antisense
