# Supplementary material for: Conservation of the S10-spc-α Locus within Otherwise Highly Plastic Genomes Provides Phylogenetic Insight into the Genus Leptospira
Source: PLoS One. 2008 Jul 16;3(7):e2752. doi: 10.1371/journal.pone.0002752 (PMC2481283; doi:10.1371/journal.pone.0002752)
Supplement: Table S3 — Amplification through the S10-spc-α operon of Leptospira spp. Positive and negative PCR scores for amplification reactions along the locus from various strains. (0.05 MB DOC) [file pone.0002752.s004.doc]

**Table S3. Amplification through the *S10-spc-α* operon of *Leptospira* spp.**

| **Species** | **Strain** | **Binary Amplification Signature*** |
| --- | --- | --- |
| *L. interrogans* | Lai | 1 1 1 1 1 1 1 1 1 1 1 1 1 1 1 1 1 1 1 1 1 1 1 1 1 1 1 1 1 1 1 1 1 1 1 1 1 1 1 1 |
| *L. interrogans* | M20 | 1 1 1 1 1 1 1 1 1 1 1 1 1 1 1 1 1 1 1 1 1 1 1 1 1 1 1 1 1 1 1 1 1 1 1 1 1 1 1 1 |
| *L. interrogans* | RGA | 1 1 1 1 1 1 1 1 1 1 1 1 1 1 1 1 1 1 1 1 1 1 1 1 1 1 1 1 1 1 1 1 1 1 1 1 1 1 1 1 |
| *L. interrogans* | Hond Utrech IV | 1 1 1 1 1 1 1 1 1 1 1 1 1 1 1 1 1 1 1 1 1 1 1 1 1 1 1 1 1 1 1 1 1 1 1 1 1 1 1 1 |
| *L. interrogans* | Pomona | 1 1 1 1 1 1 1 1 1 1 1 1 1 1 1 1 1 1 1 1 1 1 1 1 1 1 1 1 1 1 1 1 1 1 1 1 1 1 1 1 |
| *L. interrogans* | Hardjoprajitno | 1 1 1 1 1 1 1 1 1 1 1 1 1 1 1 1 1 1 1 1 1 1 1 1 1 1 1 1 1 1 1 1 1 1 1 1 1 1 1 1 |
| *L.borgpetersenii* | Lely 607 | 0 0 0 0 0 0 1 1 1 1 1 1 0 1 1 1 1 1 0 1 1 1 1 1 0 0 0 0 0 1 1 1 1 1 1 1 1 1 1 0 |
| *L.santarosai* | CZ 299 | 0 0 0 0 0 0 1 1 1 0 0 1 0 1 1 1 1 1 0 0 0 1 1 0 0 0 0 1 0 1 1 1 1 0 1 0 0 1 0 0 |
| *L. santarosai* | 1342 K | 1 0 0 0 0 0 1 1 1 0 0 0 0 1 1 0 1 1 1 0 0 1 0 0 1 0 0 0 0 1 1 1 1 0 0 0 0 1 0 0 |
| *L.noguchii* | 1161 U | 0 0 0 1 0 0 1 1 0 1 1 0 0 1 1 1 1 1 0 1 1 1 0 1 0 0 1 1 0 1 1 1 1 1 1 1 0 1 1 0 |
| *L.noguchii* | CZ 214 K | 1 0 0 0 0 0 1 1 1 1 1 0 0 1 0 0 1 1 0 1 1 1 1 1 0 0 1 1 0 0 1 1 1 1 0 0 0 1 1 0 |
| *L.weili* | Sarmin | 0 0 0 0 0 0 1 1 1 1 1 1 0 1 1 1 1 1 0 0 1 1 1 1 0 0 0 1 1 1 1 1 1 1 1 0 0 1 0 0 |
| *L.weili* | Celledoni | 1 1 0 0 0 0 1 1 0 0 0 0 0 0 1 0 1 1 1 0 0 1 0 0 0 1 0 0 0 1 1 1 1 1 1 0 0 1 0 0 |
| *L.kirschneri* | 5621 | 0 0 1 0 0 0 1 1 1 1 1 0 0 1 1 0 1 1 1 1 1 1 1 1 1 0 1 1 1 1 1 1 1 1 1 1 1 1 1 0 |
| *L.alexanderi* | A85 | 0 0 0 0 0 0 1 0 0 1 0 1 0 1 1 1 1 1 0 0 0 1 1 0 0 1 0 1 0 1 1 1 1 1 1 0 0 1 0 0 |
| *L.alexanderi* | A23 | 1 1 0 0 0 1 1 1 1 1 1 1 1 1 1 0 1 1 1 0 0 1 0 0 1 0 0 1 0 1 1 1 1 0 1 0 0 1 1 0 |
| *L.faine* | BUT 6 | 1 0 0 0 0 0 1 0 0 0 0 0 0 0 0 0 0 0 0 1 0 0 0 0 0 0 0 0 0 0 1 0 1 0 1 0 0 0 0 0 |
| *L.meyeri* | Veldrat Semarang 173 | 1 0 0 0 0 0 0 0 0 1 0 0 0 0 0 0 0 0 0 0 0 0 0 1 0 0 0 0 0 0 1 1 1 0 0 0 0 0 0 0 |
| *L.meyeri* | ICF | 1 0 0 0 0 0 1 1 0 1 1 0 1 1 1 0 1 1 1 1 0 1 1 1 1 1 0 0 1 1 1 1 1 0 1 0 0 1 0 0 |
| *L.inadai* | 10 | 0 0 0 0 0 0 0 0 1 0 0 0 0 0 0 0 0 0 0 0 0 0 0 0 0 0 0 0 0 1 1 0 1 0 0 0 0 0 0 0 |
| *L.inadai* | H6 | 1 1 1 1 1 1 1 1 1 1 1 1 1 1 1 1 1 1 1 1 1 1 1 1 1 1 1 1 1 1 1 1 1 1 1 1 1 1 1 1 |
| *L.biflexa* | Patoc I | 1 1 0 0 0 0 1 1 1 1 0 0 0 0 0 0 0 0 1 1 0 1 0 0 1 0 0 0 0 0 0 1 1 1 0 0 0 1 0 0 |

* Signature position correspond to the PCR fragments 4, 5, 6, 7, 9, 10, 11, 13, 16, 17, 18, 20, 22, 23, 24, 26, 27, 28, 31, 32, 33, 34, 35, 36, 41, 42, 43, 45, 47, 49, 51, 52, 55, 57, 58, 59, 60, 62, 65 and 66, respectably from Table 1.

Correctly sized amplicons as deduced from the positions of the primer pairs on the S10-spc-α locus are scored “1”. Anomalous products or absence of products are scored as “0”.
